# Inhibition of DPP-IV Activity and Stimulation of GLP-1 Release by Gastrointestinally Digested Black Soldier Fly Prepupae

**DOI:** 10.3390/foods12102027

**Published:** 2023-05-17

**Authors:** Anna Valentina Luparelli, Alba Miguéns-Gómez, Anna Ardévol, Stefano Sforza, Augusta Caligiani, Montserrat Pinent

**Affiliations:** 1 Department of Food and Drug, University of Parma, Parco Area delle Scienze 49/a, 43124 Parma, Italy; annavalentina.luparelli@unipr.it (A.V.L.); stefano.sforza@unipr.it (S.S.); 2 MoBioFood Research Group, Department de Bioquímica i Biotecnologia, Universitat Rovira i Virgili, c/Marcel·lí Domingo n 1, 43007 Tarragona, Spain; alba.miguens@urv.cat (A.M.-G.); montserrat.pinent@urv.cat (M.P.)

**Keywords:** edible insects, dietary protein, in vitro digestion, enterohormones, fermentation

## Abstract

The beneficial effects of an insect-based diet on human health and, in particular, the regulatory ability of digested insects’ proteins on the glycaemic response in humans are topics that need to be investigated deeper. In this work, we performed an in vitro study on the modulatory activity of gastrointestinal digested black soldier fly (BSF) prepupae on the enterohormone GLP-1 and its natural inhibitor, DPP-IV. We verified whether actions intended to valorise the starting insect biomass, i.e., insect-optimised growth substrates and prior fermentation, can positively impact human health. Our results highlight that the digested BSF proteins from all the prepupae samples had a high stimulatory and inhibitory ability on the GLP-1 secretion and the DPP-IV enzyme in the human GLUTag cell line. Gastrointestinal digestion significantly improved the DPP-IV inhibitory capacity of the whole insect protein. Moreover, it was seen that optimised diets or fermentation processes preceding the digestion, in any case, did not positively affect the efficacy of the answer. BSF was already considered one of the edible insects more suitable for human consumption for its optimal nutritional profile. The BSF bioactivity here shown, after simulated digestion, on glycaemic control systems makes this species even more promising.

## 1. Introduction

The problem of the relevant and constantly growing world population, predicted to reach 9.7 billion people during the next 30 years, raised the need to find an alternative source of high biological value nutrients, especially proteins, for human and animal requirements [1]. In this context, insects are considered a strategic solution for positive environmental and human health effects. Insect large-scale rearing is far more sustainable than livestock farming due to insect high feed conversion efficiency, limited water and land requirement, and low gas emissions [2]. Moreover, most insects, including, for instance, mealworms, buffalo worms, silkworms, crickets, and grasshoppers, are excellent sources of important fatty acids (such as linoleic, omega-6 and alpha-linolenic acids, omega-3), chitin, vitamins, minerals and proteins of high biological value [3,4,5]. The protein content in the insect body can reach 70% on a dry basis, higher than most plants and other commercial animal protein sources such as meat, eggs etc. [6,7]. Among the order of diphthera (*Stratiomyidae*), *Hermetia illucens*, also called the Black soldier fly, is one of the most promising choices for massive breeding to be used as the basis of food and feed ingredients. Numerous studies demonstrated how black soldier fly larvae/prepupae satisfy all specific dietary requirements for humans and farmed animals thanks to their optimal nutritional composition [8,9,10]. Black soldier fly prepupa, the insect life stage most studied from a nutritional point of view, has been reported to contain (expressed on dry mass) 32% of proteins, 37% of lipids, 19% of minerals and 9% of chitin [11]. Since eating insects is thought to be one of the potential answers to the growing protein world demand, the interest in understanding the effects of this alternative food source on health is increasing. The literature shows that most studies only examine the beneficial effects of an insect-based diet on livestock health [12]. For example, numerous studies showed the good support that the integration of *H*. *illucens* prepupae in a diet could have on chickens’ and other animals’ growth and gut health [12,13,14,15]. On the contrary, the studies aimed to measure the health outcomes of human subjects from insect consumption are still limited [16]. Few studies showed that their consumption could, in general, promote both human and animal health, preventing or controlling health risks such as hypertension or diabetes and reinforcing the immune system [17,18,19]. Nowakowski et al., 2020 provide a list of studies from the literature reporting the possible health benefits of whole insects or insect isolates in animal or human trials. One of these works, for example, shows the improved immune response in chickens following a *T. molitor* larvae diet [20]. Another example is reduced blood glucose and LDL cholesterol levels, and increased anti-oxidative activities were observed in diabetic mices fed with a diet with *Gryllus bimaculatus* supplements [21]. Recent research stated that some of these beneficial effects are possible because bioactive peptides from insect protein fractions can be generated [22,23,24,25]. However, the interaction of these molecules with the gastrointestinal tract and the systemic health benefits associated are topics that have not yet been adequately explored. One of the most important findings of the last years is that enzymatic hydrolysis can generate, from insect proteins, proactive peptide fragments that positively affect the postprandial glycemic response [26,27,28,29]. From in vitro and in vivo experiments, it was observed that the hydrolysate of these novel foods, as, for instance, simulated gastrointestinally digested *Alphitobius diaperinus*, can stimulate, more than other protein sources, the gut secretion of a class of hormones called incretins, including the GLP-1, that promote the insulin release and the intestinal absorption of glucose, blocking the glucagon release [30]. Moreover, they are found to be natural inhibitors of the dipeptidyl-peptidase IV (DPP-IV), a potent antagonist of GLP-1 enterohormone [26,31,32]. Faced with this evidence, it may be assumed that edible insects, if introduced into a human diet and subjected to a complete digestion process, could be an optimal source of bioactive peptides that, thanks to their mechanism of action, could play an important role in preventing and controlling metabolic diseases such as diabetes. However, more controlled studies are fundamental to confront and confirm these insect-derived ingredients’ health benefits and better assess the activity of bioactive peptides potentially generated from the digestion [30,31]. No specific investigations have yet been made concerning the black soldier fly. Thus, to evaluate more in-depth the beneficial effects of being waged by an insect-based diet on human health and in the face of the lack of information on the effects of a specific interaction between the black soldier fly species and a human intestinal model, the aim of this work was double: at first explore the modulatory activity of the digested black soldier fly on the enterohormone GLP-1 and its natural inhibitor, DPP-IV. Then, check if this effect can be affected by the insect diet or a previous fermentation process applied to the insect biomass.

## 2. Materials and Methods

### 2.1. Insect Samples and Proximate Composition

The Black soldier flies (*Hermetia illucens*, HI) prepupae samples, from larvae purchased from CIMI srl (Cuneo, Italy), derived from a larval rearing experiment carried out by the Applied Entomology laboratory of the University of Modena and Reggio Emilia (Italy), and from a fermentation, experiment carried out by the microbiologist laboratory of the university of Parma (Italy). More specifically, for the current investigation, were used: (a) Black soldier fly prepupae reared on a control diet composed of 50% wheat bran, 30% alfalfa meal and 20% corn meal (CP); (b) black soldier fly prepupae reared on an optimal agri-food by-products mixture (40% minced tomato, 30% bran and 40% ground green beans) in terms of breeding performances (PAgri) (c) black soldier fly prepupae reared on control diet, incubated with two different *Lactobacillus* (LAB) strains, LAB *rhamnosus* (PFR) and LAB *plantarum* (PFP). All the insects were killed by freezing at −20 °C because this is currently the most widespread killing method applied by insect-producing companies. Insects were then finely ground for 2 min with an IKA A10 laboratory grinder (IKA Werke GmbH & Co. KG, Staufen, Germany). The samples undergoing fermentation were prepared as described by Hadj Saadoun et al., 2020. Once ground, prepupae were blended with sugar (8.5% *w*/*w*). The mix obtained was sterilized in an autoclave at 121° for 20 min in a glass jar. 30 g of BSF prepupae/sugar substrate were then collected and incubated with a final concentration of 7 Log CFU/g of each bacterial suspension for 72 h at 30 °C for *L. plantarum* 285 and 37 °C for *L. rhamnosus* 1473. All the insect samples were lyophilized and stored at −20 °C until the analysis [33,34,35]. Standard procedures (AOAC, 2002) were used to evaluate the composition of BSF prepupae samples’ moisture, lipid and ash. Moisture was determined in the oven at 105 °C for 24 h. An automated Soxhlet extractor (SER 148/3 VELP SCIENTIFICA, Usmate Velate, Italy) was used to determine crude fat, using diethyl ether as an extraction solvent. Ashes were determined after mineralization at 550 °C for 10 h (5 h + 5 h). Total amino acids determination was carried out following the method described by Fuso et al., 2021. Briefly, 0.5 g of BSF prepupae was hydrolysed with 6 mL of HCl 6 N at 110 °C for 23 h. After cooling, the internal standard (7.5 mL of 5 mM Norleucine in HCl 0.1 M) was added. Cysteine was determined as cysteic acid after performic acid oxidation followed by acid hydrolysis. Total tryptophan was determined following the protocol proposed by Delgado-Andrade et al., 2006 with some modifications [36]. The hydrolysed samples were analysed by ultra-performance liquid chromatography with electrospray ionization and mass spectrometry detector (UPLC/ESI-MS, WATERS ACQUITY) after derivatization with 6-aminoquinolyl-N-hydroxysuccinimidyl carbamate (AQC). From the sum of total amino acids, prepupae total protein content was calculated. Chitin and other compounds’ content were calculated by the difference in the quantity of proteins, ashes, and lipids.

### 2.2. Chemicals

All the chemicals used were of analytical grade. Sodium bicarbonate, α-Amylase (EC 3.2.1.1), Porcine Pepsin (EC 3.4.23.1), Porcine pancreatic lipase (EC 3.1.1.3), and Bile salts were provided by Sigma Aldrich, Co. (St. Louis, MO, USA). Pancreatin contains enzymatic components, including trypsin, amylase, lipase, ribonuclease, and protease. The ELISA kit for total GLP-1 (catalog no. EZGLPT1-36K) was purchased from Millipore (Billerica, MA, USA). We used it according to the instructions provided by producers that could be easily obtained on the websites of each enterprise with detailed references. Gly-Pro-7- amido-4-methyl coumarin hydrobromide (Gly-Pro-AMC) was obtained from Bachem AG (Bubendorf, Switzerland). Diprotin A (Ile-Pro-Ile) was supplied by Enzo Life Sciences International (New York, NY, USA. Lactate Dehydrogenase Assay (LDH) kit was provided by QCA (Tarragona, Spain). All the other solvents, salts, acids, and bases were purchased from Sigma-Aldrich.

### 2.3. Simulated Gastrointestinal Digestion

The Black soldier fly prepupa samples were subjected to an in vitro gastrointestinal digestion, adapted from the INFOGEST harmonized protocol, as previously described by Miguéns-Gómez et al., 2020. Briefly, the different prepupa samples were collected, the groups still whole and finely ground at the moment, and mixed sequentially with three different simulated digestive fluids (salivary, gastric, and intestinal), each composed of a stock solution with a specific concentration of electrolytes, enzymes, CaCl_2_ and water, to simulate the three corresponding typical phases of a whole digestion process. At first, to reproduce the oral phase, 10 g of each prepupa was directly added to the salivary electrolytic stock solution in a ratio of 50/50 *w*/*v*. The mixtures were mixed for 2–5 min using Ultra-Turrax T25 homogenizer (IKA Werke, Staufen, Germany). Once these appeared homogeneous, the α-Amylase (EC 3.2.1.1) was added to achieve 75 U/mL, CaCl_2_ (final concentration: 0.75 mM), and water. The mixture was adjusted at pH 7 and remixed at 37 °C for two minutes. After this, a specific volume of gastric electrolytic solution was added to reach a ratio of salivary mixture to gastric fluids of 50/50 *v*/*v*. To this second phase, Porcine trypsin (EC 3.4.21.4), to achieve 2000 U/mL, CaCl_2_ (final concentration: 0.075 mM) and water were added. After adjusting the pH to 3, the gastric phase was agitated at 37 °C for 120 min. For the last phase, the gastric phase volume was mixed with the same amount of simulated Intestinal fluid (50:50 *v*/*v*). Porcine pancreatic lipase (EC 3.1.1.3) to achieve 100 U/mL, Bile salt mixture (10 mM), CaCl_2_ (final concentration: 0.3 mM) and water were added. The pH was further adjusted to 7, and the final solution was maintained in agitation at 37 °C for another 120 min. Consequently, digested black soldier flies’ samples were heated to 90 °C for 30 min to stop the enzymatic reactions and centrifuged for 5 min, at 4000 rpm and at 4 °C to separate the indigested part. The supernatants were collected, lyophilized, and stored at −20 °C until use. As enzymes control, the same procedures were applied to an additional sample without inserting food to digest at the beginning of the process.

### 2.4. Characterization of Digestion Products by SDS-PAGE

After the artificial digestion, a Bicinchoninic Acid (BCA) Assay for protein quantification of the digested, negative controls and enzyme control samples was carried out using a BCA kit (Pierce, Thermo Fisher Scientific, Waltham, MA, USA) (Walker, J. M. 2009). An SDS-PAGE was performed to attest to the successful digestion of the treated samples. The electrophoresis was carried out by mixing 30 µL of each digested, undigested and enzyme control sample with a protein concentration of 2 μg/μL with 10 microliters of 4× sample loading buffer (125 mM Tris HCl (pH 6.8), 2.5% (*w*/*v*) sodium dodecyl sulfate (SDS), 0.1% (*w*/*v*) bromophenol blue, 25% (*v*/*v*) glycerol, 25% (*v*/*v*) β-mercaptoethanol). After heating the mixture at 100 °C for 5 min, 15 µL from each sample was collected and loaded on a 16% polyacrylamide gel. A molecular weight marker (Page Ruler, Thermo Fisher Scientific) was included on each gel. Gels were then stained with colloidal Coomassie Blue (Bio-Rad Laboratories, Hercules, CA, USA).

### 2.5. GLUTag Cell Line Culture

The GLUTag cells used in the present work were kindly donated by Prof. Staels (University Lille, Institut Pasteur de Lille, Lille, France) with permission of Prof. Drucker (Lunenfeld-Tanenbaum Research Insitute, Toronto, ON, Canada). The medium where the cells were cultured was composed of 88% of DMEM (Dulbecco’s modified Eagle’s medium) containing 1 g/L D-glucose, supplemented with 10% foetal bovine serum (Sigma-Aldrich, Madrid, Spain), 1% of 100 U/mL/100 mg/L Penicillin/Streptomycin and 1% of Glutamine (final concentration 2 mM) (Lonza, O Porriño, Spain). The cells were incubated under a 5% CO_2_-humidified atmosphere at 37 °C. At least three GLUTag cell passages were cultivated consequentially to allow each treatment to have adequate biological replicates for the GLP-1 secretion test.

### 2.6. GLP-1 Secretion Test

GLUTag cells were plated onto 24-well plates precoated with Matrigel (Lonza, O Porriño, Spain) at a density of 200.000 cells/mL 24 h before the secretion study. Cells were washed twice with PBS buffer and treated for 2h at 37 °C with the CP, PFR, PFP, and Pagri samples following intestinal digestion dissolved in HEPES buffer (1.25 mM) at a concentration of 5 mg protein/mL. All the treatments were performed by duplicate in each cell plate and repeated for 3 passages. After the treatment, the medium of each well was collected and stored at −80 °C in aliquots of 25 µL until the determination of total GLP-1 and LDH release assay following the manufacturer’s instructions. Then the cells were lysed with RIPA buffer, lysates were stored at −80 °C, and then used to analyse total protein content using a BCA kit (Pierce, Thermo Fisher Scientific) and for LDH quantification in the cells.

### 2.7. Determination of the DPP-IV Inhibitory Activity (% Inhibition and IC_50_)

The potential inhibition of the black soldier fly samples before and after gastrointestinal digestion on DPP-IV activity was measured using 96-well microplates, according to the method used by Casanova-Martí et al., 2019, with slight modifications [37]. The inhibition assay consisted in dissolving directly in the microplate 15 µL of DPP-IV enzyme (to reach 0.26 mU per well) and 10 µL sample containing 6 mg of proteins/mL per well (0.06 mg protein per sample) with 25 µL of 100 mM Tris·HCl assay buffer (pH 8.0). The microplate was pre-incubated for 10 min at 37 °C. Then, the assay was started by adding 50 µL of the chromogenic substrate H-Gly-Pro-AMC (final concentration 0.01 mM). The plate containing the mixtures was read in a microplate reader at Ex:380 nm/Em:460 nm at 37 °C each minute for 30 min. As a reference inhibitor and positive control of the assay was used Diprotin A (Ile-Pro-Ile) was well-known to inhibit the DPP-IV enzyme. A blank sample (with no insect end enzyme) and an enzyme sample (enzymes with no insect) were also tested respectively as negative and enzyme control. Each sample was tested in triplicate. The DPP-IV inhibition activity of each sample was expressed as a percentage derived from the difference between the enzyme activity in the presence of the test samples and the enzyme control sample’s activity. Then, to calculate the IC_50_ (the concentration of the sample required to cause 50% inhibition of the enzyme activity), the DPP-IV inhibition experiment was performed again, using five different concentrations (ranging from 15 to 60 and from 3 to 0.5 mg of protein/mL respectively for the no digested and digested) of the previously tested control prepupa samples. The IC50 values, expressed by the estimated peptide concentration (μg/mL) and volume (μL), were determined by plotting the percentage of inhibition as a function of the test hydrolysate concentration using GraphPad prism v4.0 for windows (Dotmatics, Boston, MA, USA).

### 2.8. Data Analysis

The data are expressed as the mean ± SD or ±standard error and result from at least a triplicate analysis of independent samples. Differences between the means of the tested groups of samples were determined using a one-way ANOVA test, followed by Tukey’s post-hoc test. Statistics were performed using the SPSS software (SPSS, Chicago, IL, USA). Only *p*-values < 0.05 were considered significant.

## 3. Results

### 3.1. Characterization of the Samples

The content of lipids, proteins, ashes and other compounds, including chitin of BSF prepupae, was evaluated to assess potential variations in the nutrient distribution of the samples based on the different rearing or fermentation conditions. The complete proximate composition is reported in Table 1.

The control prepupa (CP) has the highest percentage of lipids and ashes, present in significatively lower concentrations in the samples undergoing different rearing conditions and fermentation with the two different LAB strains. The significantly higher content of ‘other compounds’ in PFR and PEP is essentially due to the sugar addiction to the insect substrates at the moment of LAB incubation to trigger the fermentative process. Because of the nature of the investigation presented in the following paragraphs, special attention shall be given to the protein content of the samples. As shown in Table 1, protein content ranged from 32.2 to 36.7 g/100g of dry matter, with no significant differences among the samples.

### 3.2. SDS-PAGE Analysis of Digested and Undigested Samples

**Figure 1:**
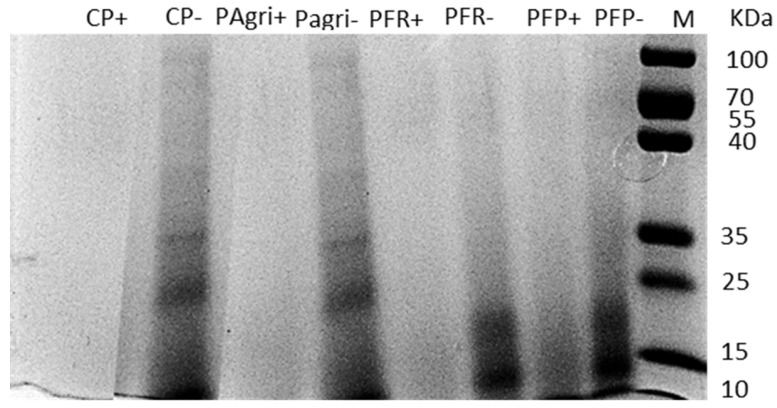
Protein hydrolysis after black soldier fly prepupae intestinal digestion. The digested sample is indicated with “+”, while the undigested controls are marked with “−”. The protein load was adjusted in each lane to 30 μg of protein. A molecular weight marker (10–180 kDa) was included. CP, control prepupa; Pagri, prepupa reared on an optimal agri-food by-products mixture; PFR, prepupa fermented with LAB rhamnosus; PFP, prepupa fermented with LAB plantarum; M, molecular weight marker.

To verify proper hydrolysis during the in vitro incubation of prepupae samples with digestive proteases, digests were analysed by SDS–PAGE electrophoresis. The results are shown in Figure 1, where digested, and samples before digestion are compared. The SDS-PAGE analysis of the undigested CP and PAgri showed a similar protein pattern, the richest bands with a higher molecular mass. On the other hand, PFR and PFP samples presented all the protein bands concentred in a lower molecular weight range (25–10 kDa), probably due to the previous fermentation process [35]. In any case, there was a clear difference between the protein pattern of the digested (+) and non-digested (−) samples. In the non-digested samples, it is possible to observe the presence of protein bands which completely disappeared in the digested samples, showing that the digestive protocol, hydrolysing the proteins and generating small peptides not visible with this kind of analysis, degraded the initially present proteins. Furthermore, even the previously fermented samples (PFR and PFP) showed fainter bands after simulated gastrointestinal digestion, suggesting a further degradation to smaller peptides.

### 3.3. Black Soldier Fly Protein Has DPP-IV Inhibitory Activity

**Figure 2:**
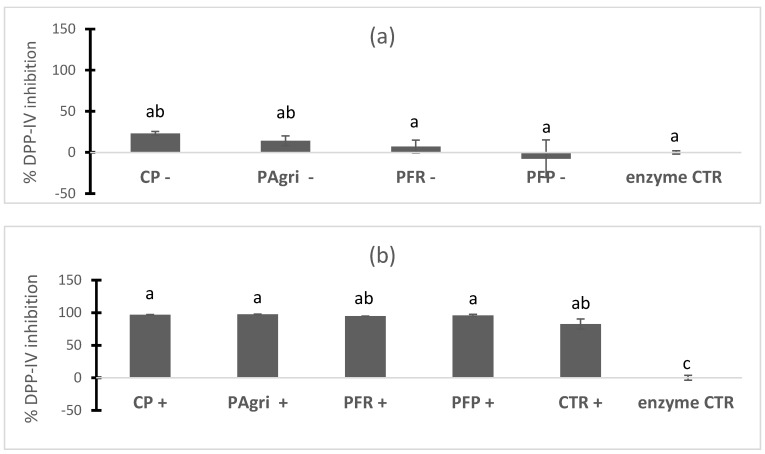
DPP-IV inhibitory activity of in vitro undigested (─) (**a**) and digested (+) (**b**) BSF prepupae, respectively, compared with the enzyme control (enzyme CTR) and the positive inhibitory control, Diprotin A (CTR+). The per cent of DPP-IV inhibition was determined using 6 mg of protein/mL as the final assay concentration of the sample. The results are expressed as the mean ± SEM of three replicates. One-way ANOVA and Tukey’s post hoc test were used for multiple comparisons. Different letters (a,b,c) indicate significant differences (*p*-values < 0.05). CP, control prepupa; Pagri, prepupa reared on an optimal agri-food by-products mixture; PFR, prepupa fermented with LAB rhamnosus; PFP, prepupa fermented with LAB plantarum.

The first purpose of this work was to assay whether BSF protein had DPP-IV inhibitory capacity and to determine whether optimized rearing conditions or previous fermentation of the BSF would affect it. Figure 2a shows that BSF CP and PAgri undigested samples had a slight but significant DPP-IV inhibitory capacity. This was similar between the two samples. Surprisingly, the fermentation of BSF with two different *Lactobacillus* strains did not increase but decreased the DPP-IV inhibitory activity of BSF. Then the effects of the same samples submitted to an in vitro gastrointestinal digestion was tested. The results are shown in Figure 2b. It can be observed that the simulated intestinal digestion produced hydrolysates with very high inhibitory activity (more than 90%) against the DPP-IV enzyme. Such inhibition was even higher than the positive control Diprotin A (CTR+) response at the tested conditions (82% inhibition). Negative control using only the gastrointestinal digestion vehicle without insects sample was also carried out at the different concentrations, showing no effects on DPP-IV activity (results not shown). As well as with the undigested samples, the BSF had no significant differences depending on the diet conditions. After gastrointestinal digestion, fermented samples (PFR and PFP) showed the same DPP-IV inhibitory activity as non-fermented samples.

To better quantify the effects of simulated gastrointestinal digestion on the DPP-IV activity of BSF protein, the IC50 was calculated for the CP protein before and after the digestion. Table 2 shows that the digested CP proteins showed a very low IC50 value, 10 times lower than the IC50 value of the undigested CP.

### 3.4. Intestinal Digests of BSF Prepupa Stimulate GLP-1 Secretion

As a further step, the potentiality of BSF prepupae to activate at the intestinal level the GLP-1 secretion was evaluated in vitro by using GLUTag cells. The enteroendocrine cell line was incubated for one hour with 5mg protein/mL of the four samples submitted to simulated gastrointestinal digestion to better mimic what might occur in the intestine.

First, we ruled out a potential cytotoxic effect of the treatments on the GLUTag cell line through LDH assay. The results obtained (Table 3) showed that the percentage of LDH released to the medium was very low (<3% in all cases), discarding toxic effects.

**Figure 3:**
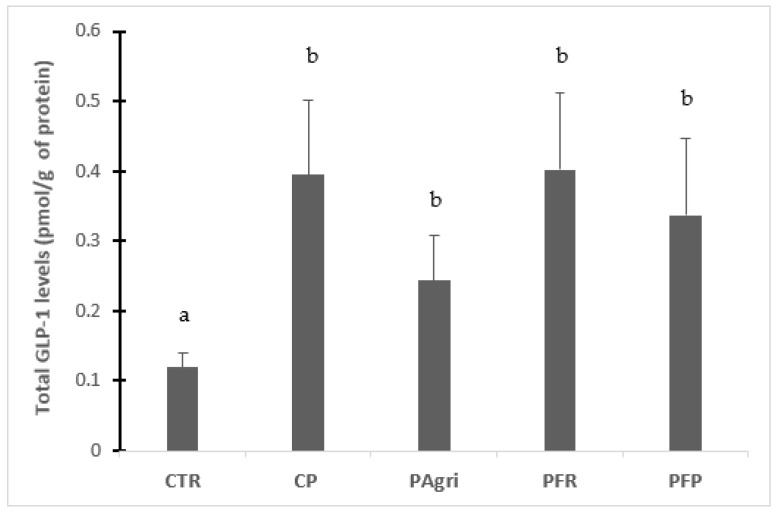
Effect of gastrointestinal digested prepupa samples on total GLP-1 secretion in GLUTag cells. Cells were incubated with 5 mg protein/mL of the different samples for 30 min at 37 °C, and total GLP-1 release to the medium was assessed. Values were normalised by protein content in each well. Results are presented as the mean ± SEM. The sample number was n = 6 from three different passages. One-way ANOVA and Tukey’s post hoc test were used for multiple comparisons. Other letters (a,b) indicate significant differences (*p*-values < 0.05). CTR, HEPES buffer; CP, control prepupa; Pagri, prepupa reared on an optimal agri-food by-products mixture; PFR, prepupa fermented with LAB rhamnosus; PFP, prepupa fermented with LAB plantarum.

Figure 3 shows the total GLP-1 release to the medium after treatment with the different BSF samples. All the gastrointestinal digested samples led to a significant increase in the cell secretion of total GLP-1 compared to untreated cells. On the contrary, there are no statistical differences among the different BFS prepupae samples. Previous incubation of the prepupae with *Lactobacillus rhamnosus* or *L. plantarum* does not modify the GLP-1- secretory capacity of the gastrointestinally digested samples.

## 4. Discussion

The present investigation aimed to evaluate in vitro the potential intestinal response in humans after ingesting BSF prepupa samples, focusing on their potential to inhibit the DPP-IV enzyme and stimulate GLP-1 hormone release. Furthermore, we aimed to compare whether this potential intestinal response was modulated by the diet in which the prepupae were reared and to test if a prepupae fermentation could improve them with two different *Lactobacillus* strains.

The DPP-IV enzyme is intensely involved in regulating the glucose level in the bloodstream. Its central role is to rapidly inactivate the incretins (GLP-1), avoiding in a healthy subject a hypoglycemic effect [38]. The increased incidence of human diseases such as diabetes, which is linked to a hyperglycemic condition, is leading to a constant search for new functional food that can prevent it, reducing the use of pharmacological approaches [39]. In this context, one of the most investigated aspects is the ability of some protein sources to produce, after digestion, bioactive peptides with DPP-IV inhibitory activity. In this paper, we show that BSF proteins have DPP-IV inhibitory capacity for the first time, and the BSF digestion led to an enormously increased inhibitory capacity. Even if there is no literature on the bioactivity following an in vitro digestion of the BSF proteins, similar investigations related to other edible insects allowed some comparisons [31,32,40,41]. Our results on DPP-IV inhibitory activity can be considered in agreement with similar research on species as *Alphitobius diaperinus* or *Gryllodes sigillatus* [31,41], in which antidiabetic peptides production was observed and attributed to the action of enzymes as pepsin, known to cut proteins in appropriate positions to generate fragments exerting DPP-IV inhibitory activity [31]. In fact, in works in which the bioactivity of edible insects on the intestine is tested, a proper enzymatic hydrolytic pre-treatment is necessary to obtain sufficiently high yields of peptides, the main responsible for the most important biological activities [24,41,42]. A slight inhibitory activity against the enzyme DPP-IV has also been found in the undigested prepupa, especially in CP and PAgri samples. This can be explained by the high concentration of proteins within the insect biomasses and consequently by the presence of a potential endogenous enzymatic activity that can trigger even before the proteolytic treatments an initial break of insect proteins and a small production of bioactive peptides, generating inhibitory activity [31]. Different peptides have been shown to act through other DPP-IV inhibitory mechanisms, including competitive, uncompetitive, non-competitive and mixed-type modes. Further characterization of our samples will help elucidate the specific mechanisms used to exert such inhibition [28].

Regarding the IC50 of these digests, data on BSF species were unavailable. If compared with data on different food protein sources, the IC50 value of the BSF prepupa can be considered lower, suggesting a higher concentration in insects of inhibitory peptides [22,37]. In the Casanova-Martí et al., 2019 study, the value of chicken feet hydrolysates was around 30 mg estimated protein per mL. In another study, the DPP-IV inhibitory potency of simulated gastrointestinal digested hemp, pea, rice and soy were evaluated, and IC50 values found ranged from 1.85 ± 0.34 to 4.50 ± 0.55 mg dw hydrolysate mL^−1^ [22]. Besides this, the IC50 found in our samples was lower and respected the IC50 values observed for other insect hydrolysates. From in vitro digested *A. diaperinus* proteins, for instance, Lacroix et al., 2019 found an IC50 value of around 1.0 mg/mL, while Nongonierma et al., 2018 reported an IC50 ranging from 0.40 to 1.0 mg/mL for crickets [31,32]. This makes the black soldier fly, among the insect species investigated, one of the best candidates as an alternative functional ingredient to the most common protein sources.

BSF prepupae digested protein was also tested for its ability to stimulate the enterohormone GLP-1 in an in vitro system. GLP-1 is a kind of hormone belonging to the class of incretins and is secreted from intestinal L-cell in response to meal ingestion. This hormone stimulates insulin and inhibits glucagon, encouraging blood sugar balance [38,43,44,45]. The ability of some food to stimulate the secretion of this hormone compared to others gives them a particular interest as natural coadjutant to a human health condition. Hence, numerous studies in the literature aim to study how different food sources stimulate the system of incretins using other models (in vivo, ex vivo, and in vitro) [46,47,48]. For example, in the study of Mochida et al., 2010 it is shown that meat hydrolysate has a high power in stimulating GLP-1 secretion [46]. Geraedts et al., 2011 and Hall et al., 2003 found a good capacity for GLP-1 hormone secretion in casein, whey, codfish, and eggs. A dual role in inhibiting DPP-IV and enhancing GLP-1 release in vitro has been shown for some food sources, including chicken feet, cuttlefish or whey proteins [49,50,51,52,53]. Although all interest in these potentialities, information on how novel food sources, such as insects, could modulate the enterohormone release is lacking. The study of Miguéns-Gómez et al., 2020 is one of the few measuring the enterohormone secretion ex vivo in human/animal intestines treated with in vitro digestions of a specific insect *Alphitobius diaperinus*, showing how the insect, concerning other proteins sources (beef and almond), was the one of the most effective in inducing the secretion of total GLP-1 in human colon samples. The results obtained in the present study demonstrate the potential of Black soldier fly prepupae to increase GLP-1. In our investigation, the GLP-1 values obtained from the BSF prepupae treatments (0.51 ± 0.06 to 0.58 ± 0.10 pg/mL) were higher than the insect species studied in the work cited above (0.19 ± 0.04 pg/mL). However, it is essential to remember that many variables can affect the results related to the secretory ability of GLP-1 from BSF species. For instance, this investigation’s results were obtained using an in vitro intestinal model. The effect in vivo could be different. Real digestion conditions, the bioavailability of black soldier fly-derived peptides, the intestinal portion where they are absorbed, and the proximity with the cells that release incretins are all factors that could affect the antidiabetic potential of the insect base product and should be adequately studied. Even if these results could be influenced by many factors, including insect species, digestion process conditions, and interaction model used (in vitro in GLUtag cells vs. ex vivo), the GLP-1 response to the digested BSF prepupae suggests that this insect, among the species currently investigated, presents a strong potential beneficial power in reduce glycemia. Moreover, future studies would also be interesting to assess the adverse effects or possible risks (such as allergic reactions) of consuming insects as a regular food source, especially in Western countries, where their consumption is not typical.

Another goal of the present study was to investigate whether rearing BSF prepupae on different agri-food by-products concerning the control diet would modify the effects on the gastrointestinal tract of BSF. The specific by-products (40% minced tomato, 30% bran and 40% ground green beans) on which the PAgri was reared led to an improvement in terms of breeding performances (number of larvae and growth rate) concerning the prepupae grown on the control diet (CP) [33]. Our results show that changes in the diet did not lead to significant changes in the BSF properties since the results observed resembled those of the commercially fed insects. Furthermore, CP and PAgri showed similar DPP-IV inhibitory capacity, mainly when gastrointestinally digested, and there were no differences in the levels of GLP-1-induced secretion by the digested samples. These results suggest that the protein and peptides contained in the prepupae were similar. This assumption is supported by the data obtained by proximate composition, which revealed that no significant changes were present in the protein content of the insects according to the diet administered. A similar condition has been observed in the work of Fuso et al. (2021), where it was shown how prepupa grown on cereals and vegetable by-products, a diet similar to the one used for BSF sample PAgri, had no significant differences in the protein content concerning the prepupa reared on control diet [34]. However, an in-depth analysis of the peptide profile of gastric and intestinal in vitro digestions is needed to understand better the results obtained with these in vitro experiments.

In the present study, the effects of BSF fermentation using two strains of *Lactobacillus* were also evaluated. Our results showed that the fermentation process broke down the BSF protein into smaller peptides since, as expected, the undigested BSF samples (CP and PAgri) presented more bands (Figure 1) than the undigested fermented samples (PFR and PFP). Supposedly, the fermentation process leads to a pre-digestion of the food, affecting the protein fraction and generating smaller peptides [54,55]. Fermented and not digested BSF samples showed bands in the part of the gel concerning proteins of minor size, ranging between 10 to 25 kDa. This result is entirely in line with other investigations, where the protein bands of fermented food samples were also observed to be in the same range [56,57]. Although the high potential of *Lactobacillus* cultures for trigging a substrate’s pre-digestion during fermentation and producing bioactive peptides able to prevent hyperglycaemia was already proven [58], in this study, it was not observed that an improved DPP-IV inhibitory activity derived from incubation of BSF prepupa samples with selected LAB strains. When these samples were subjected to simulated gastrointestinal digestion, they underwent further cleavage, as observed in the electrophoresis gel. The DPP-IV inhibitory activity reached the same levels as the digested non-fermented BSF, suggesting that the final released peptides did not significantly differ. These results contrast with another study that showed how a fermentative pre-treatment followed by an in vitro digestion led to a decreased antihyperglycemic activity compared to a digested non-fermented substrate [59]. Moreover, Nongonierma et al., 2018 noted a decreased DPP-IV inhibitory activity following the simulated in vitro digestion of a fermented cricket protein, suggesting that the potential bioactive peptides released by the bacterial pre-digestion have been broken down during the simulated digestion. In contrast, in our case, the BSF digestion probably allowed the release of the peptides required for the DPP-IV inhibitory action. These tiny peptides were not released before fermentation. This difference is likely related to a very low degree of proteolysis that characterized our undigested fermented samples, as shown in the electrophoretic gel. The small peptides generated by fermentation are very limited in number, not probably leading to an effect on intestinal hormonal regulation. Indeed, food bioactivity derived from fermentation can depend on the microorganism species, growth substrate, time, and environment temperature, which explains why the results observed in similar studies could be different [55,58,60].

Similar results were observed regarding GLP-1 secretion. However, only the in vitro-digested samples were tested [61]. So as Nowakowski et al., 2020 occurred with DPP-IV, fermentation of the BSF did not modify the GLP-1 stimulatory effect of the in vitro digested samples, suggesting that the digestion applied shortened to the same extent the fermented and non-fermented proteins and peptides, leaving to the release of bioactive peptides independently from the previous fermentation. These amino acids and/or peptides released can activate GLP-1 release through different mechanisms. Several transporters (amino acid transporters, peptide transporter 1 (PepT1)) and receptors (the protein-coupled receptor family C group 6 subtype A (GPRC6A), the umami receptor (T1R1–T1R3 heterodimer) and the G protein-coupled receptor 92/93 (GPR92/93) have been suggested as mediators of the GLP-1 release, each one activating different signalling pathways involving intracellular calcium release that leads to GLP-1 secretion. Thus, again future characterization of our digested samples will allow a more in-depth description of the mechanisms involved [61].

## 5. Conclusions

In conclusion, the study shows a very high ability of all BSF-digested proteins to inhibit the DPP-IV enzyme and stimulate the GLP-1 secretion in GLUTag cells. Our results highlight that gastrointestinal digestion improves the DPP-IV inhibitory capacity of whole protein. Moreover, feeding the insects with a different composition diet does not modify the above-mentioned intestinal effects. We also showed that a previous fermentation with *Lactobacillus* is optional for these activities, although we cannot rule out that different fermentation protocols could strengthen them. These results suggest that the BSF is one of the edible insects more suitable for human consumption. In addition to having an optimal nutritional profile, they benefit human health. Being these preliminary tests conducted using in vitro conditions, more investigations are planned to understand if they are replicable in vivo. This is the first time the black soldier fly insect has been investigated as a functional food for its hypothetical positive impact on human metabolic problems like diabetes. The good results derived from this experiment could be an incentive to explore more in-depth the beneficial effects on the human health of novel protein sources, such as insects, which are still unexplored from numerous points of view.

## Figures and Tables

**Table 1:** Nutritional composition of BSF prepupae. Values are expressed dry matter and result from four replicate analyses.

Composition (%)	CP *	PAgri	PFR	PFP
Lipid	37.1 ± 0.1 ^a^	29.9 ± 4.3 ^b^	10.6 ± 0.3 ^c^	16.4 ± 1.5 ^c^
Proteins, from total AA	32 ± 2 ^a^	36.7 ± 0.3 ^a^	36.2 ± 0.3 ^a^	35.4 ± 0.6 ^a^
Ash	19 ± 1 ^a^	7.8 ± 2.3 ^b^	6.5 ± 0.1 ^b^	6.3 ± 0.1 ^b^
Other compounds (including chitin)	11.9 ± 1 ^a^	25.6 ± 0.7 ^b^	46.7 ± 0.3 ^c^	41.9 ± 0.6 ^d^

One-way ANOVA and Tukey’s post-hoc test were used for multiple comparisons. Different letters (^a,b,c,d^) indicate significant differences (*p*-values < 0.05). CP, control prepupa; Pagri, prepupa reared on an optimal agri-food by-products mixture; PFR, prepupa fermented with LAB rhamnosus; PFP, prepupa fermented with LAB plantarum. * Caligiani et al., 2018 [11].

**Table 2:** IC50 activity of black control soldier fly prepupa (CP) undigested (─) or submitted to simulated gastrointestinal digestion (+).

Sample	IC50 (mg/mL)
CP─	14.54 ± 0.11
CP+	0.14 ± 0.16

IC50 values are reported as the mean from triplicate assays ± SD.

**Table 3:** LDH release to the medium after 1 h of digested BSF prepupa treatments in GLUTag cells.

	CP	PAgri	PFR	PFP	Vehicle-Treated Cells
% LDH released to the medium	2.7 ± 0.6 ^a^	2.4 ± 0.6 ^ab^	2.6 ± 0.5 ^ab^	2.3 ± 0.1 ^b^	1.2 ± 1.0 ^ab^

The % was determined using medium and cells from each treatment. The results are the mean ± SEM of six replicates from three cell passages. One-way ANOVA and Tukey’s posthoc test were used for multiple comparisons. Different letters (^a,b^) indicate significant differences (*p*-values < 0.05). CP, control prepupa; Pagri, prepupa reared on an optimal agri-food by-products mixture; PFR, prepupa fermented with LAB *rhamnosus*; PFP, prepupa fermented with LAB *plantarum.*

## Data Availability

Data is contained within the article.
